# Supplementary material for: Learning curve and surgical outcome of robotic assisted colorectal surgery with ERAS program
Source: Sci Rep. 2022 Nov 29;12:20566. doi: 10.1038/s41598-022-24665-w (PMC9709162; doi:10.1038/s41598-022-24665-w)
Supplement: Supplementary file 1 — Supplementary Information 1. [file 41598_2022_24665_MOESM1_ESM.zip › LSC Raw data-Ñ[▒K/142/P_ñw╝╨░O▒Kñσ.pdf]

[redacted]

Pathologic diagnosis:
1. Sigmoid colon, laparoscopic anterior resection ---
Adenocarcinoma, moderately differentiated, arising in a tubulovillous adenoma. Tumor invades submucosa focally. The bilateral cut ends and mesocolic lymph nodes (0/14) are free of tumor.
2. IMA root, dissection --- Unremarkable lymph nodes (0/5).
3. Lymph nodes, para-aortic, biopsy --- Negative for metastasis (0/1).
4. Colon, cut end, distal, resection --- Negative for tumor.

Prognostic and predictive factor:
1. Tumor size: 2.2x2.2x2 cm.
2. Depth of invasion: Submucosa.
3. Histologic grade: G2: Moderately differentiated.
4. Angiolymphatic invasion: Not identified.
5. Perineural invasion: Not identified.
6. Tumor deposits (discontinuous extramural extension): Not identified.
7. Macroscopic tumor perforation: Not identified.
8. Surgical Margins:
a) Proximal Margin: Uninvolved by invasive carcinoma, distance of tumor from margin: 6.7 cm.
b) Distal Margin: Uninvolved by invasive carcinoma, distance of tumor from margin: 4.6 cm.
c) Radial or Mesenteric Margin: Uninvolved by invasive carcinoma, distance of tumor from margin: 4.2 cm.
9. Lymph node status: Uninvolved (0/20).
10. Extranodal involvement: Not applicable.
11. Tumor regression grading S/P CCRT: No known presurgical therapy.
12. Type of polyp in which invasive carcinoma arose: Tubulovillous adenoma.
13. Additional pathologic findings: Not identified.
14. Pathological TNM Stage: pT1N0 (According to the eighth edition, American Joint Committee on Cancer Staging Guidelines for Tumors).
15. TNM descriptors: Not applicable.

Gross description:
The specimen consists of 1) a segment of colon, 13.5 cm in length and 3.8 cm in proximal cut end circumference, 5 cm in distal circumference, and mesocolic soft tissue. A tan brown polypoid lesion, 2.2x2.2x2 cm, is noted, 4.6 cm to the distal cut end. On cross section, no definite invasion is seen. A black stitch is also seen, 2.5 cm from the proximal cut end, labeled "T1 lesion scar". No lesion is found around the stitch. The both cut ends are free of tumor grossly. 2) IMA root, a piece of tan yellow soft tissue, 2.5x1.5x1.3 cm. 3) "PA LN", a piece of yellow tan and soft tissue, 0.9x0.8x0.6 cm. 4) distal cut end, a piece of tan mucosa-covered soft tissue, 1.7x1.5x0.9 cm. Representative part for section: A) proximal cut end B) distal cut end C-E) polypoid tumor F-G) stitch side H) radial margin J) specimen 2 L) specimen 4 M-) lymph nodes; all for section: K) specimen 3 (bivalved).

Note:
This case has been peer reviewed by two doctors.
#T-59600_2 #M-82633_2 2 1105 B0000F
#T-C4560_2 #M-09183_2 0 1105 000000

已電子簽章

敬告：本單僅供醫師參考使用，不具任何申請效力！！
請病患妥為保管，若不慎遺失需自負個人資料外洩責任！
